# Detection and genomic characterization of *Salmonella* Infantis and *Klebsiella* serotypes in loggerhead sea turtles (*Caretta caretta*), Italy

**DOI:** 10.3389/fvets.2026.1827576

**Published:** 2026-05-07

**Authors:** Camilla Smoglica, Alexandra Chiaverini, Vincenzo Olivieri, Camilla Zeni, Anna Janowicz, Lisa Di Marcantonio, Emanuela Di Giulio, Anna Angrilli, Alessandra Cornacchia, Mattia Ferrara, Francesco Pomilio, Cristina Esmeralda Di Francesco

**Affiliations:** 1 Department of Veterinary Medicine, University of Teramo, Teramo, Italy; 2 Istituto Zooprofilattico Sperimentale dell'Abruzzo e del Molise “G. Caporale”, Teramo, Italy; 3 Centro Studi Cetacei Onlus (CSC), Pescara, Italy

**Keywords:** antibiotic resistance, *Klebsiella*, One Health, rehabilitation, *Salmonella*, sea turtles, whole genome sequencing

## Abstract

This study describes the detection and genomic characterization of *Salmonella enterica* serovar Infantis ST32 and previously unreported *Klebsiella* serotypes isolated from stranded *Caretta caretta* along the Italian Adriatic coast. Cloacal and fecal samples collected during rescue and rehabilitation activities yielded multiple isolates identified through MALDI-TOF MS and subjected to antimicrobial susceptibility testing and whole-genome sequencing. *Salmonella* Infantis isolates displayed a uniform ST32 profile and were recovered from a turtle at admission and from remaining positive animals prior to release. These findings suggest intermittent shedding and possible acquisition of infection during hospitalization. Genomic relatedness among isolates indicated potential intra-facility transmission. In contrast, *Klebsiella* isolates exhibited heterogeneous profiles, including several multidrug-resistant strains harboring resistance determinants of clinical relevance. The identification of novel *Klebsiella* serotypes and the first report of *S*. Infantis ST32 in loggerhead sea turtles expand current knowledge on pathogen diversity in marine reptiles and emphasize their role as sentinels for antimicrobial resistance in marine environments. Overall, these findings highlight the importance of integrating advanced diagnostic and genomic tools into wildlife health monitoring, with implications for rehabilitation practices, environmental surveillance, and zoonotic risk assessment within a One Health framework.

## 1 Introduction

The Mediterranean Sea is undergoing rapid environmental transformation driven by climate change, including unprecedented warming trends documented in recent years (1). Climate-driven environmental changes influence microbial community dynamics, pathogen persistence, and antimicrobial resistance (AMR) emergence and may therefore influence host-associated microbiomes, with potential implications for antimicrobial resistance dynamics in marine wildlife (2). The loggerhead sea turtle (*Caretta caretta*) is the most common marine turtle species in the Mediterranean Sea and has been considered an indicator of environmental health (3). Indeed, loggerhead sea turtles interact extensively with anthropogenic pollution, including wastewater, marine debris, and agricultural runoff, making them effective bioindicators of environmental contamination (4, 5). Additionally, sea turtles have been proposed as sentinel species for monitoring AMR dissemination in marine environments due to their long lifespan, wide migratory range, and close interaction with coastal environments impacted by anthropogenic activities (6, 7). The loggerhead sea turtle has therefore been the focus of several studies addressing bacterial colonization and antimicrobial resistance patterns. Early investigations documented the presence of antimicrobial-resistant Gram-negative bacteria in oral and cloacal microbiota of stranded or free-ranging *C. caretta*, highlighting resistance to commonly used antimicrobials, including beta-lactams, tetracyclines, aminoglycosides, and sulfonamides (4). Subsequent studies suggested that loggerhead sea turtles may act as carriers of resistant bacteria acquired from contaminated coastal waters, sediments, nesting beaches, and foraging areas, reinforcing their role as bioindicators of anthropogenic pressure in marine environments (6, 8). Several investigations conducted in the Mediterranean basin, particularly in Italy, reported the isolation of Enterobacterales, as well as *Aeromonas* spp., *Vibrio* spp., and other opportunistic Gram-negative bacteria from both healthy and injured turtles, with a non-negligible proportion of isolates exhibiting multidrug-resistant (MDR) phenotypes (9–11).

More recently, molecular approaches have begun to complement culture-based investigations, allowing the detection of antimicrobial resistance genes and mobile genetic elements associated with turtle-related bacterial communities (6). Whole genome sequencing (WGS) has been applied to *Listeria* isolates recovered from marine turtles, demonstrating its value in resolving species identification, virulence traits, and resistance determinants that cannot be reliably inferred using conventional methods alone (12). However, the application of high-resolution genomic tools in *Caretta caretta* remains limited. This results in significant gaps in the understanding of AMR ecology and transmission pathways at the wildlife–environment interface.

These findings raise concerns from a One Health perspective, as resistant bacteria harbored by marine wildlife may contribute to the environmental persistence and geographic dissemination of AMR determinants, potentially bridging marine, terrestrial, animal, and human health compartments.

AMR in wildlife has implications for both ecosystem and public health due to bacterial exchange across humans, animals, and the environment, particularly involving Enterobacterales such as *Salmonella* and *Klebsiella* (13, 14). *Salmonella* spp. is well-known zoonotic pathogens capable of causing a broad spectrum of diseases in both humans and animals, ranging from mild gastroenteritis to severe systemic infections (13). *Klebsiella* spp., particularly *K. pneumoniae*, in environmental reservoirs and animal hosts represents a critical public health concern due to its role as a reservoir of multidrug-resistant genes and its capacity for zoonotic transmission, thereby contributing to the spread of antibiotic-resistant infections in humans (14–16). In addition, the 2024 WHO bacterial priority pathogen list highlights Gram-negative bacteria resistant to last resource antibiotics, including *Klebsiella* and *Salmonella* species, as a critical global health concern (17).

This study provides new insights into *Salmonella* Infantis and *Klebsiella* strains isolated from *Caretta caretta* individuals found stranded or rescued along the Adriatic Sea. The research also expands investigations to an underexplored area of the Mediterranean by applying antibiotic susceptibility test (AST) and advanced methodologies such as Matrix-Assisted Laser Desorption/Ionization Time-Of-Flight Mass Spectrometry (MALDI-TOF) and Whole Genome Sequencing (WGS) to characterize the strains both phenotypically and genotypically.

## 2 Materials and methods

Sea turtles were rescued along the Adriatic coast from January to June 2023 by the “Luigi Cagnolaro” Cetacean Study Center in Italy. Sample types included cloacal swabs, feces, and cutaneous swabs collected at admission (T0) and prior to release (T1). Microbiological culture used Buffered Peptone Water as non-selective enrichment for 24 h at 37 °C followed by subculture using streak plating technique on MacConkey agar (Liofilchem, Italy) at 37 °C for 18–24 h. The detection of *Salmonella* spp. was performed by enrichment and plating out on selective media. Specifically, 100 μl of non-selective enrichment were incubated in 10 ml Rappaport Vassiliadis broth (Liofilchem, Italy) at 41 °C for 24 h. Afterward, aliquots of cultures were spread in Xylose Lysine Deoxycholate agar (Liofilchem, Italy), and the plates were incubated at 37 °C for 24–48 h.

Isolates were identified using MALDI-TOF MS (Bruker, UK). AST was performed using Sensititre GNX2F plate (Thermo Scientific™, Lenexa, KS, United States), interpreted using European Committee on Antimicrobial Susceptibility Testing (EUCAST) (18) breakpoints and Clinical and Laboratory Standards Institute (CLSI) (19) where EUCAST breakpoints were unavailable. Genomic DNA extraction and Illumina sequencing followed standardized protocols (20, 21) and metadata are available at Bioproject accession number PRJNA1295198. *Klebsiella* taxonomy was assigned using Pathogenwatch (22), and AMR genes were detected with ResFinder (23). *Salmonella* isolates were analyzed alongside 249 additional Italian *Salmonella* Infantis strains obtained from the SRA repository using core genome MLST (cgMLST). The analysis utilized the *Salmonella enterica* task template, which incorporates 3,002 core gene targets based on the EnteroBase *S. enterica* cgMLST v2 scheme (20).

## 3 Results

The bacterial strains isolated in this study derived from a total of 11 cloacal and cutaneous swabs and fecal samples collected from 7 loggerhead sea turtles (Table 1). The cutaneous swabs were collected in a case of periarticular wound with necrosis area as reported in Table 1. Seven *Klebsiella oxytoca*, and four *Salmonella* spp. were identified by MALDI-TOF. Among these, five *Klebsiella* strains were isolated from T0 samples of three different turtles, and the remaining two strains came from one turtle at the moment of the release. Salmonella strains were recovered from four different turtles, starting from T1 samples except for one isolated coming from T0 sample.

**Table 1:** Details of animals (ID, sex, CCL, holding tank shared), region of stranded site, therapy used during recovery time and types of collected samples.

Animal ID	Sex^*^	CCL	Region of stranded site	Sample types	Therapy	Number of holding tank	Recovery time (days)
297	NA	39.9	Abruzzo	Feces (T0)	None	1	Not released
302	NA	35.7	Abruzzo	Cloacal swab (T0)	None	1	Not released
303	NA	33.5	Molise	Cloacal swab (T0), periarticular lesion swab (T0), necrosis area swab (T0)	Enrofloxacin 10 mg/kg q 24 h 7 gg	–	Not released
307	F	80.7	Abruzzo	Feces (T0), Cloacal swab (T1)	None	2	45
308	M	76.8	Abruzzo	Cloacal swab (T1)	None	2	40
310	F	76	Abruzzo	Cloacal swab (T1)	None	3	36
315	NA	62	Abruzzo	Cloacal swab (T1)	None	3	43

^*^NA, not applicable because sex was undetermined for certain individuals.

The *Salmonella* strains showed susceptibility to all tested antibiotics while all *Klebsiella* strains resulted to be resistant to at least one antibiotic and five isolates were multidrug resistant (Table 2).

**Table 2:** Details of Minimum Inhibitory Concentration (MIC) values of antibiotic susceptibility tests.

MALDI-TOF bacterial species	ID Animal	Sample type	MIC																				
			AMI	TIM2	AZT	P/T4	SXT	GEN	FEP	TOB	LEVO	DOX^*^	CIP	MIN^*^	MERO	FOT	TGC	ETP	IMI	DOR	COL	POL	TAZ
*Kl. oxytoca°*	297	Feces (T0)	**32**	16/2	**16**	**32/4**	4/76	**4**	**16**	**4**	**4**	**16**	**1**	2	1	1	0.25	0.25	< 1	0.12	0.25	0.25	< 1
*Kl. oxytoca°*	302	Cloacal swab (T0)	**32**	< 16/2	**8**	< 8/4	< 0.5/ 9.5	< 1	4	< 1	**2**	< 2	< 0.25	< 2	< 1	< 1	< 0.25	**1**	**8**	0.12	< 0.25	2	< 1
*Kl. oxytoca°*	303	Cloacal swab (T0)	< 4	< 16/2	**8**	**16/4**	2/38	< 1	**8**	< 1	**4**	2	< 0.25	2	< 1	**16**	2	< 0.25	< 1	< 0.12	< 0.25	< 0.25	**8**
*Kl. oxytoca*	303	Periarticular lesion swab (T0)	< 4	< 16/2	4	< 8/4	1/19	< 1	4	< 1	**2**	2	0.5	4	< 1	< 1	0.25	< 0.25	< 1	< 0.12	0.5	0.5	< 1
*Kl. oxytoca°*	303	Necrosis area (T0)	< 4	< 16/2	**8**	**32/4**	2/38	< 1	4	< 1	**2**	8	0.5	< 2	< 1	1	0.25	< 0.25	< 1	< 0.12	0.25	< 0.25	4
*Kl. oxytoca°*	307	Cloacal swab (T1)	**32**	**64/2**	**16**	**32/4**	4/76	**8**	4	**8**	**2**	**16**	0.5	2	1	< 1	0.25	< 0.25	< 1	0.12	< 0.25	0.25	< 1
*Kl. oxytoca°*	307	Cloacal swab (T1)	**16**	< 16/2	4	**64/4**	1/19	**8**	4	**8**	1	2	0.25	2	1	< 1	0.25	< 0.25	2	< 0.12	0.25	< 0.25	< 1
*S*. Infantis	307	Feces(T0)	< 4	< 16/2	< 2	< 8/4	< 0.5/ 9.5	< 1	< 2	< 1	< 1	2	< 0.25	2	< 1	< 1	0.25	< 0.25	< 1	< 0.12	0.5	0.5	< 1
*S*. Infantis	308	Cloacal swab (T1)	< 4	< 16/2	2	< 8/4	0.5/ 9.5	< 1	2	< 1	1	2	0.25	2	< 1	< 1	0.25	< 0.25	< 1	< 0.12	0.5	0.5	< 1
*S*. Infantis	310	Cloacal swab (T1)	< 4	< 16/2	< 2	< 8/4	< 0.5/ 9.5	< 1	< 2	< 1	< 1	2	< 0.25	2	< 1	< 1	0.25	< 0.25	< 1	< 0.12	0.25	0.5	< 1
*S*. Infantis	315	Cloacal swab (T1)	< 4	< 16/2	2	< 8/4	0.5/ 9.5	< 1	< 2	< 1	< 1	2	< 0.25	2	< 1	< 1	< 0.25	< 0.25	< 1	< 0.12	0.25	0.5	< 1

AMI, Amikacin; TIM2, Ticarcillin / Clavulanic Acid constant 2; AZT, Aztreonam; P/T4, Piperacillin / Tazobactam constant 4; SXT, Trimethoprim / Sulfamethoxazole; GEN, Gentamicin; FEP, Cefepime; TOB, Tobramycin; LEVO, Levofloxacin; DOX, Doxycycline; CIP, Ciprofloxacin; MIN, Minocycline; MERO, Meropenem; FOT, Cefotaxime; TGC, Tigecycline; ETP, Ertapenem; IMI, Imipenem; DOR, Doripenem; COL, Colistin; POL, Polymixin B; TAZ, Ceftazidime.

MIC values for resistant isolates, based on EUCAST breakpoints (18), are shown in bold.

Asterisks (^*^) indicate antibiotics evaluated using CLSI breakpoints (19).

Circles (°) denote multidrug-resistant isolates.

T0, samples collected at time of admission to recovery center. T1, sample collected before the release back to the natural environment.

Bacterial species determination using PathogenWatch platform results in 4 *Klebsiella grimontii*, 2 *Klebsiella michiganensis*, and 1 *Klebsiella oxytoca*. Chromosomal resistance genes, as *aph(3*′*)-Ia* and *blaOXY*, were detected as reported in Table 3.

**Table 3:** Whole Genome Sequencing characterization of *Klebsiella* strains.

Animal ID	Pathogenwatch bacterial species	Sequence type	Sample type	Aminoglycosides	Chromosomal class A beta-lactamase
297	*K. oxytoca°*	101	Feces(T0)	–	blaOXY-2
302	*K. grimontii°*	^*^34d	Cloacal swab (T0)	–	blaOXY-6-3
303	*K. grimontii*	^*^8d53	Cloacal swab (T0)	–	blaOXY-6-2
303	*K. grimontii*	^*^8d53	Periarticular lesion swab (T0)	–	blaOXY-6-2
303	*K. grimontii°*	^*^8d53	Necrosis area (T0)	–	blaOXY-6-2
307	*K. michiganensis°*	^*^1df9	Cloacal swab (T1)	aph3′)-Ia	blaOXY-1-7
307	*K. michiganensis*	^*^1df9	Cloacal swab (T1)	aph3′)-Ia	blaOXY-1-7
307	*S*. Infantis	34	Feces(T0)	acc(6′)-Iaa	–
308	*S*. Infantis	34	Cloacal swab (T1)	acc(6′)-Iaa	–
310	*S*. Infantis	34	Cloacal swab (T1)	acc(6′)-Iaa	–
315	*S*. Infantis	34	Cloacal swab (T1)	acc(6′)-Iaa	–

Circles (°) denote multidrug-resistant isolates identified by antibiotic susceptibility test.

Asterisks (^*^) indicate new sequence types.

**Figure 1:**
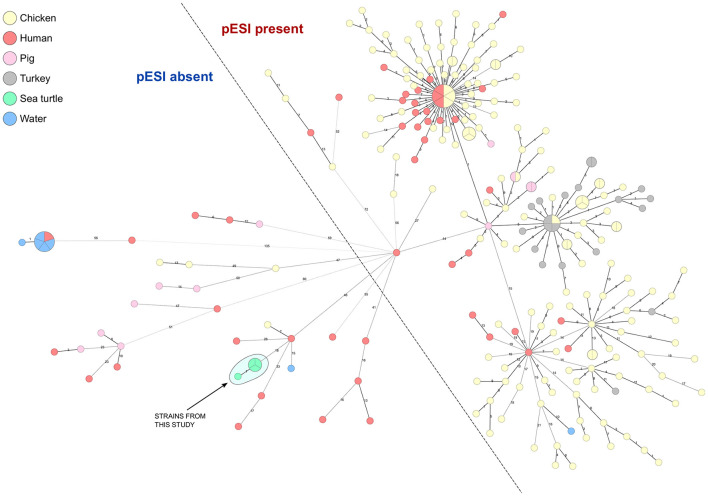
Minimum spanning tree (MST) of *Salmonella* Infantis in Italy. A set of 253 genomes, including 4 strains from this study and 249 published available sequences, were analyzed using cgMLST. The MST was generated based on 3,002 core genome target genes, with missing values ignored. The broken line separates strains that harbor pESI megaplasmid from the strains where this plasmid was not detected.

The analysis of four *Salmonella* isolates showed *Salmonella* Infantis sequence type 32 and no detection of any plasmid and AMR genes, with the exception of the cryptic gene aac(6′)-Iaa detected in all isolates. Clustering analysis revealed that all *Salmonella* Infantis genomes from sea turtles were closely related, with only one differing by a single core genome allele. The closest related genomes identified were from human, chicken, and water sources, differing from this study's strains by 18, 19, and 21 alleles, respectively (Figure 1).

## 4 Discussion

The methodologies applied in this study significantly contributed to the detailed characterization of bacterial strains isolated from loggerhead sea turtles. The existing literature on bacterial infections and antimicrobial resistance (AMR) in *Caretta caretta* largely relies on culture-based identification and phenotypic antimicrobial susceptibility testing, often without advanced molecular confirmation, thus limiting the resolution of epidemiological and phylogenetic inferences (24). In this context, the combined use of MALDI-TOF MS and WGS represents a substantial methodological improvement, allowing accurate species identification and comprehensive characterization of resistance determinants, particularly within the *Klebsiella oxytoca* complex (*KoC*).

Notably, the existing literature rarely employs advanced techniques such as MALDI-TOF and WGS for the identification of bacterial isolates in loggerhead sea turtles, limiting the ability to draw meaningful epidemiological conclusions (24). Indeed, WGS analysis revealed a notable discordance between MALDI-TOF identification and genomic species assignment, leading to the detection of *Klebsiella grimontii* and *Klebsiella michiganensis*, which are frequently misidentified as *K. oxytoca* using routine diagnostic tools. Indeed, the genome-based taxonomy recognizes *K. oxytoca* as a heterogeneous complex comprising multiple species, including the aforementioned species, belonging to different phylogroups of the complex (38). This finding underscores the importance of genomic approaches for reliable identification of closely related *Klebsiella* species and for a more accurate assessment of their epidemiological and clinical relevance in wildlife hosts.

In humans, bacteria of *KoC* are components of oral and intestinal microflora, but they are able to cause several health care infections (e.g., hemorrhagic colitis and meningitis), particularly in immunocompromised patients (25, 26). In addition, *KoC* bacteria are emerging as pathogens causing severe infections in companion animals, and subclinical or occasionally mastitis in livestock (27, 28). Recently, they were described also in shellfish and goldfish (29).

The clinical relevance of each species of the *KoC* bacteria, including the colonization, prevalence as pathogens in various infections, the disease spectrum and severity, remains largely overlooked (38). However, it may represent a main concern considering that members of the *Klebsiella* genus are recognized among priority bacterial pathogens of public health importance due to their capacity to acquire and disseminate resistance to critically important antimicrobials (17).

*Klebsiella* isolates in this study displayed a higher level of antimicrobial resistance, with several strains classified as multidrug resistant. The detection of chromosomally encoded resistance genes, including *blaOXY* variants and *aph(3*′*)-Ia*, highlights the role of environmental and wildlife-associated *Klebsiella* as potential reservoirs and indicators of resistance determinants for domestic animals and humans. The detection of MDR *Klebsiella* strains, including resistant isolates to WHO Critically Important Antimicrobials (17), raises One Health concern for AMR dissemination in coastal ecosystems and rehabilitation centers. As previously reported by other authors, the MDR *Klebsiella* strains in wildlife suggest multiple epidemiological links between human-related environments, domestic animals and wild fauna (30). Within this framework, marine ecosystem may be considered a critical hotspot to collect and maintain resistant bacteria relevant to public and animal health and originating from contaminated coastal waters, sediments, and wastewater discharges (31–33). Sea turtles may acquire resistant bacteria through exposure to this environment and, once admitted to rehabilitation facilities, additional opportunities for bacteria circulation may arise among animals, technical staff, and the hospitalization environment (7, 34, 35).

Contrary to what was observed for Klebsiella strains, all *Salmonella enterica* serovar Infantis isolates resulted susceptible to the tested antimicrobials and lacked acquired resistance genes, with the exception of the cryptic aminoglycoside resistance gene *aac(6*′*)-Iaa*.

*Salmonella enterica* serovar Infantis is currently the fourth most frequently reported serovar in human infections and the most common serovar identified in the broiler production chain (36). Previous study realized on Italian *S*. Infantis isolates highlights an increase detection of ESBL-producing *S*. Infantis attributed to an expansion if the psESI megaplasmid (20). The absence of pESI megaplasmid and major AMR genes in *S*. Infantis isolates collected in this study contrasts with aforementioned dominant Italian lineages mainly associated to food-chain (20). Additionally, the sea turtle isolates did not cluster closely with any of the publicly available Italian strains, suggesting a different ecological origin.

Clustering patterns and temporal detection of Salmonella isolates suggest a possible intra-facility transmission, which warrants improved biosecurity measures. In this respect, the rehabilitation centers can be considered a critical hub for pathogens dissemination and exposure to technical staff, due to the high turn-over of turtles recovered coming from different geographical areas, with little or no anamnestic and clinical information (34, 37). In order to reduce the transmission of pathogens and antibiotic resistant bacteria the adequate biosecurity actions should include a quarantine tank for turtles at admission, application of diagnostic tools to evaluate the sanitary state of animals, use of personal protective equipments, and disinfection of instruments and surfaces.

A limitation of the study is the relatively small number of loggerhead sea turtles sampled. Therefore, the findings should be interpreted with caution, as they may not be fully representative of the broader free-ranging loggerhead sea turtle population or other rehabilitation settings. Nevertheless, these results provide preliminary evidence of the occurrence and genomic features of *S*. Infanis and *Klebsiella* spp. in this host and support the need of larger multicenter studies to better asses their epidemiological significance.

## 5 Conclusions

Consistent with previous studies (4, 6), this study reinforces the concept that loggerhead sea turtles can act as sentinels for AMR in marine environments and highlights the importance of integrating wildlife into antimicrobial resistance surveillance frameworks. The application of genomic tools in this context not only improves bacterial identification and resistance profiling but also provides valuable insights into the ecology and epidemiology of AMR at the human–animal–environment interface.

## Data Availability

The data presented in the study are deposited in the NCBI Bioproject repository, accession number PRJNA1295198.
